# Nonlinear relationship of red blood cell indices (MCH, MCHC, and MCV) with all-cause and cardiovascular mortality: A cohort study in U.S. adults

**DOI:** 10.1371/journal.pone.0307609

**Published:** 2024-08-02

**Authors:** Dan Li, Aiting Wang, Yeting Li, Zhishen Ruan, Hengyi Zhao, Jing Li, Qing Zhang, Bo Wu

**Affiliations:** 1 The First Clinical College, Shandong University of Traditional Chinese Medicine, Ji Nan, People’s Republic of China; 2 Dongying People’s Hospital, Dongying, People’s Republic of China; 3 Department of Cardiovascular Medicine, The First Affiliated Hospital of Shandong University of Traditional Chinese Medicine, Jinan, People’s Republic of China; 4 The First Affiliated Hospital of Shandong First Medical University, Jinan, People’s Republic of China; The First Hospital of Jilin University, CHINA

## Abstract

**Background:**

In recent years, increasing attention has been focused on the impact of red blood cell indices (RCIs) on disease prognosis. We aimed to investigate the association of mean corpuscular hemoglobin (MCH), mean corpuscular hemoglobin concentration (MCHC), and mean corpuscular volume (MCV) with mortality.

**Methods:**

The study used cohort data from U.S. adults who participated in the 1999–2008 National Health and Nutrition Examination Survey. All-cause mortality was the primary outcome during follow-up, with secondary cardiovascular mortality outcomes. COX regression was applied to analyze the connection between RCIs and mortality. We adopted three models to minimize potential bias. Smooth-fit curves and threshold effect analyses were utilized to observe the dose-response relationship between RCIs and all-cause and cardiovascular mortality. In addition, we performed sensitivity analyses.

**Results:**

21,203 individuals were enrolled in our research. During an average 166.2 ± 54.4 months follow-up, 24.4% of the population died. Curve fitting indicated a U-shaped relationship between MCV and MCH with all-cause mortality, and the relationship of MCHC to all-cause mortality is L-shaped. We identified inflection points in the relationship between MCV, MCH, and MCHC and all-cause mortality as 88.56732 fl, 30.22054 pg, 34.34624 g/dl (MCV <88.56732 fl, adjusted HR 0.99, 95 CI% 0.97–1.00; MCV >88.56732 fl, adjusted HR 1.05, 95 CI% 1.04–1.06. MCH <30.22054 pg, adjusted HR 0.95, 95 CI% 0.92–0.98; MCH >30.22054 pg, adjusted HR 1.08, 95 CI% 1.04–1.12. MCHC <34.34624 g/dl, adjusted HR 0.88, 95 CI% 0.83–0.93). Besides, the MCV curve was U-shaped in cardiovascular mortality (MCV <88.56732 fl, adjusted HR 0.97, 95 CI% 0.94–1.00; MCV >88.56732 fl, adjusted HR 1.04, 95 CI% 1.01–1.06).

**Conclusion:**

This cohort study demonstrated that RCIs (MCH, MCHC, and MCV) were correlated with mortality in the general population. Three RCIs were nonlinearly correlated with all-cause mortality. In addition, there were nonlinear relationships between MCH and MCV and cardiovascular mortality.

## Introduction

Recently, there has been increasing concern about the adverse effects of various red cell indices (RCIs) on patient symptoms and fatality outcomes. Common RCIs include red blood cell distribution width (RDW), hemoglobin (Hb), and hematocrit (HCT). RDW, a novel indicator of inflammation, is not only an independent predictor of mortality in coronary heart disease and chronic heart failure [1, 2], but also correlates with mortality in non-cardiovascular patients [3–5]. Abnormal Hb levels have been shown to be associate with the occurrence and prognosis of cerebral hemorrhage, cardiovascular disease, and worsening cognitive impairment [6–8]. HCT is correlated with survival in infectious diseases as well as chronic cardiac and renal diseases [9–11]. In addition, studies on the RCIs ratio class (Hb-RDW ratio, RDW-albumin ratio) are emerging [12–14].

Mean corpuscular hemoglobin (MCH), mean corpuscular hemoglobin concentration (MCHC), and mean corpuscular volume (MCV), which also belong to RCIs, seem to have been neglected. They are commonly utilized in the clinic as discriminatory indicators of anemia type. MCV classifies anemia as macrocytic, normocytic, and microcytic [15]. Studies typically analyze MCV in conjunction with RDW (RDW-CV). For instance, the inclusion of MCV strengthened the correlation between RDW and hypertension-induced target organ damage and helped to increase the value of RDW in predicting survival prognosis [16, 17]. The mean hemoglobin level in red blood cells is defined as MCHC, which is a proxy for the amount of oxygen-carrying information [18, 19]. MCHC is obtained by dividing MCH with MCV [20].

To date, few studies have analyzed the relationship between MCH, MCHC, MCV, and death risk, especially in the general population. Therefore, we planned to apply the data from the National Health and Nutrition Examination Survey (NHANES) to investigate such relations.

## Methods

### Study participants

The NHANES was sponsored by the Centers for Disease Control and Prevention (CDC) for data collection on a biennial cycle beginning in 1999–2000. The collection of data consisted of physical examinations, biospecimens, and standardized household interviews. The NHANES utilizes a stratified, multi-stage probability sampling methodology to select a nationally representative sample from the United States population [21]. Data regarding the study can be accessed on the NHANES website (www.cdc.gov/nchs/nhanes/index.htm). The National Center approved NHANES for Health Statistics Research Ethics Review Board. The data used were de-identified. All information from the NHANES program is available and free for public, so the agreement of the medical ethics committee board was not necessary.

**Fig 1:**
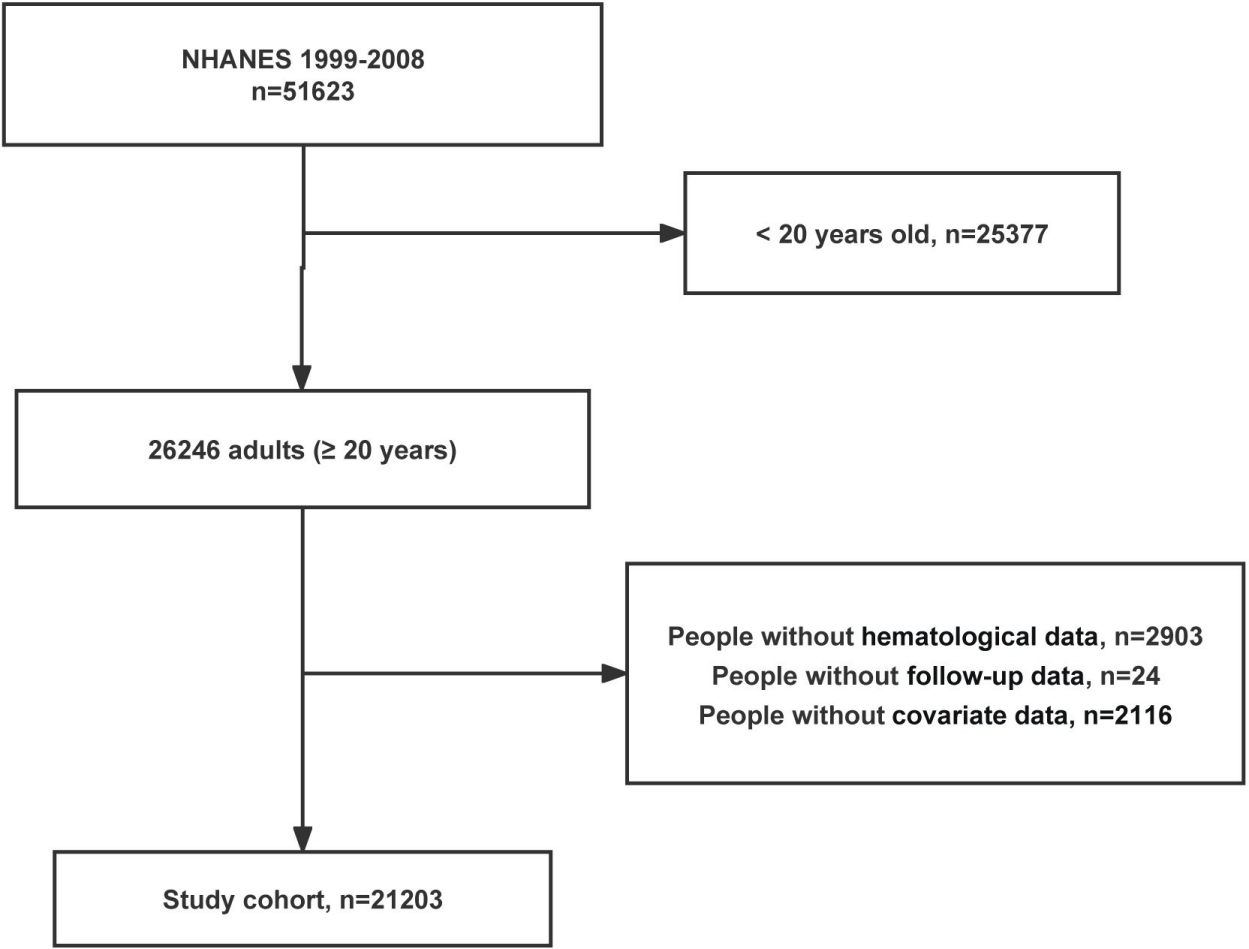
Flow chart.

The survey included data for five cycles from 1999–2008. After excluding participants <20 years and those with missing RCIs data, 21,203 individuals were ultimately enrolled in this study. The specific exclusion process was depicted in Fig 1.

### Data collection

RCIs (MCH, MCHC, and MCV) were measured by the NHANES laboratory affiliated with the CDC. We incorporated demographics (gender, race, age, body mass index [BMI], education, and smoking status) derived from self-reported data. The race was divided into four mutually exclusive categories: Non-Hispanic White, Non-Hispanic Black, Mexican American, and others. BMI was categorized into three classes (<25, 25–30, and >30 kg/m^2^). Determine the level of education of participants by asking for the highest level of the school from which they graduated (less than high school diploma, high school degree, college and beyond). We classified smoking status as never smoked (less than 100 cigarettes in a lifetime), previously smoked (more than 100 cigarettes but has quit), and current smoker.

In addition, the patient’s self-reported medical history (cardiovascular disease [CVD], hypertension, hyperlipidemia, diabetes, chronic obstructive pulmonary disease [COPD], chronic kidney disease [CKD], cancer, and anemia) was considered comorbidities. The diagnostic criteria for the above comorbidities are detailed in the additional document.

### Determination of mortality

Mortality follow-up was derived from death data tracked by the National Death Index, which has been updated through December 31, 2019. All-cause mortality was the primary outcome during follow-up, with secondary outcomes of cardiovascular mortality. We categorized the causes of death by the International Classification of Diseases (ICD)-10. Cardiovascular-related deaths are defined using codes I00-I09, I11, I13, I20-I51 (heart disease) and I60-69 (stroke).

### Statistical analysis

Analysis of variance for continuous variables and the χ^2^ test were adopted to compare the distribution of age, race, BMI, RCIs, and comorbidities between genders. After grouping the RCIs into quintiles, the distribution of population characteristics between groups was observed separately. We examined the relationship between RCIs and all-cause mortality with weighted COX regression. Furthermore, we adjusted for potential bias through three models. Model I adjusted for age and sex. Model II adjusted for age, sex, race, BMI, smoking history, and education. Model III adjusted for Model II plus hypertension, hyperlipidemia, diabetes, CVD, COPD, CKD, cancer, and anemia (Table 1). Smooth-fit curves were utilized to investigate whether there were linear dose-response relationships between RCIs and all-cause and cardiovascular mortality. And smooth-fit curves after gender grouping were done. Threshold effect analyses were performed based on it. We performed sensitivity analyses. First, because of the correlation between RCIs and anemia, we performed the analysis again after excluding anemia. Second, we excluded deaths that occurred two years before follow-up to minimize potential reverse causality [22]. And subgroup analyses of the relationship between the three RCIs and all-cause mortality were conducted.

**Table 1:** Model expression formula.

Model	Formula
I	Age + sex
II	Age + sex + race + BMI + smoking history + education
III	Age + sex + race + BMI + smoking history + education + hypertension + hyperlipidemia + diabetes + CVD + COPD + CKD + cancer + anemia

The statistically significant difference was standardized as a two-sided P value <0.05. Based on the sampling weighting documentation provided by NHANES, we performed a regression analysis based on the "wtmec2yr" and "wtmec4yr" weights. The statistical analyses were performed with R v4.1.3 (http://www.R-project.org, The R Foundation).

## Results

### Baseline characteristics

Table 2 displayed a slightly higher proportion of males than females (50.4% vs 49.6%). Compared to male, female participants were older, more educated, and less likely to smoke. The mean values of MCV, MCH, and MCHC among the participants were 89.8 ± 5.6 fl, 30.5 ± 2.2 pg, and 33.9 ± 0.9 g/dl, respectively. Furthermore, three RCIs were generally higher in males than in females. Combined hypertension, CKD, cancer and anemia are more likely in women. There were 24.4% deaths during an average follow-up time of 166.2 ± 54.4 months. A higher proportion of male deaths in all-cause and cardiovascular mortality.

**Table 2:** Baseline characteristic of the study population.

Variables	Total (n = 21203)	Female (n = 10524)	Male (n = 10679)	*P* value
**Age, years**	50.5 ± 18.3	50.7 ± 18.3	50.2 ± 18.4	0.040
**Ethnicity, %**				0.017
Non-Hispanic White	10634 (50.2)	5186 (49.3)	5448 (51.0)	
Mexican American	4424 (20.9)	2189 (20.8)	2235 (20.9)	
Non-Hispanic Black	4155 (19.6)	2115 (20.1)	2040 (19.1)	
Other Race	1990 (9.4)	1034 (9.8)	956 (9.0)	
**Education, %**				< 0.001
<High school diploma	3060 (14.4)	1430 (13.6)	1630 (15.3)	
Completed high school	8585 (40.5)	4255 (40.4)	4330 (40.5)	
≥ College	9558 (45.1)	4839 (46.0)	4719 (44.2)	
**BMI, %**				< 0.001
< 25	6553 (30.9)	3443 (32.7)	3110 (29.1)	
25–30	7535 (35.5)	3158 (30.0)	4377 (41.0)	
> 30	7115 (33.6)	3923 (37.3)	3192 (29.9)	
**Smoke, %**				< 0.001
Never smoker	10831 (51.1)	6396 (60.8)	4435 (41.5)	
Former smoker	5584 (26.3)	2136 (20.3)	3448 (32.3)	
Current smoker	4788 (22.6)	1992 (18.9)	2796 (26.2)	
**RCIs**				
MCV, fl	89.8 ± 5.6	89.1 ± 5.9	90.4 ± 5.2	< 0.001
MCH, pg	30.5 ± 2.2	30.2 ± 2.4	30.7 ± 2.1	< 0.001
MCHC, g/dl	33.9 ± 0.9	33.9 ± 0.9	34.0 ± 0.9	< 0.001
**Comorbidities, %**				
CVD	2462 (11.6)	1068 (10.1)	1394 (13.1)	< 0.001
Hypertension	8901 (42.0)	4520 (42.9)	4381 (41.0)	0.005
Hyperlipidemia	5541 (26.1)	2590 (24.6)	2951 (27.6)	< 0.001
Diabetes	3256 (15.4)	1570 (14.9)	1686 (15.8)	0.079
CKD	4139 (19.5)	2185 (20.8)	1954 (18.3)	< 0.001
COPD	838 (4.0)	355 (3.4)	483 (4.5)	< 0.001
Cancer	1895 (8.9)	995 (9.5)	900 (8.4)	0.009
Anemia	1531 (7.2)	967 (9.2)	564 (5.3)	< 0.001
**Follow-up time, months**	166.2 ± 54.4	169.2 ± 52.1	163.2 ± 56.5	< 0.001
**Mortality, %**				
All-cause	5173 (24.4)	2307 (21.9)	2866 (26.8)	< 0.001
Cardiovascular	1349 (6.4)	588 (5.6)	761 (7.1)	< 0.001

BMI: body mass index; RCIs: red blood cell indices; MCV: mean corpuscular volume; MCH: mean corpuscular hemoglobin; MCHC: mean corpuscular hemoglobin concentration; CVD: cardiovascular disease; CKD: chronic kidney disease; COPD: chronic obstructive pulmonary disease.

In addition, we demonstrated the basic characteristics of the population after dividing quintiles for the three RCIs (S1–S3 Tables). In these five groups, the three indicators were characterized by the highest number of females in the lowest quintile and the highest proportion of males in the highest quintile. In terms of MCH and MCV, those in the highest quintile had the maximum all-cause and cardiovascular mortality, while in MCHC, those in the lowest quintile group suffered the highest number of all-cause and cardiovascular deaths.

### Association between RCIs and all-cause mortality

When RCIs were regarded as continuous variables, the risk of death was elevated by 2% for each unit increase in MCV and MCH. Mortality was reduced by 6% for each unit increase in MCHC (Table 3). In the COX regression, the control groups of the three quintile RCIs were their respective Q3 groups. After adjusting for confounding, the HRs for all-cause mortality in the Q1 (≤29.1 pg) and Q5 (≥32.2 pg) groups of MCH were 1.18 (95% CI: 1.05–1.32, *P* = 0.004), 1.24 (95% CI: 1.14–1.35, *P* <0.001). In contrast to the Q3 (33.8–34.2 g/dl) group of MCHC, all-cause mortality was 22% higher in the Q1 (≤33.3 g/dl) group. The Q2 (33.4–33.7 g/dl) group had a 16% higher risk of death. The HRs for the lowest and highest quintiles in the MCV group are 1.14 (95% CI: 1.02–1.28, *P* = 0.027) and 1.33 (95% CI: 1.21–1.46, *P* <0.001), respectively.

**Table 3:** The relationship between RCIs and all-cause mortality.

	Continuous, HR (95% CI), *P*	Q1, HR (95% CI), *P*	Q2, HR (95% CI), *P*	Q3	Q4, HR (95% CI), *P*	Q5, HR (95% CI), *P*
**MCH (pg)**		≤29.1	29.2–30.3	30.4–31.1	31.2–32.1	≥32.2
Model I	**1.02 (1.00, 1.04)** **0.041**	**1.30 (1.16, 1.46)** **<0.001**	1.05 (0.95, 1.16) 0.32	1 (ref)	0.99 (0.89, 1.11) 0.92	**1.36 (1.23, 1.50)** **<0.001**
Model II	**1.03 (1.01, 1.05)** **0.003**	**1.32 (1.18, 1.48)** **<0.001**	1.05 (0.95, 1.16) 0.34	1 (ref)	0.97 (0.88, 1.08) 0.63	**1.26 (1.16, 1.38)** **<0.001**
Model III	**1.02 (1.01, 1.04)** **0.012**	**1.18 (1.05, 1.32)** **0.004**	1.02 (0.92, 1.13) 0.65	1 (ref)	0.99 (0.89, 1.10) 0.84	**1.24 (1.14, 1.35)** **<0.001**
**MCHC (g/dl)**		≤33.3	33.4–33.7	33.8–34.2	34.3–34.7	≥34.8
Model I	**0.93 (0.88, 0.98)** **0.004**	**1.27 (1.16, 1.38)** **<0.001**	**1.16 (1.06, 1.27)** **0.002**	1 (ref)	1.09 (0.99, 1.20) 0.07	**1.13 (1.02, 1.26)** **0.018**
Model II	**0.93 (0.88, 0.97)** **0.002**	**1.26 (1.15, 1.38)** **<0.001**	**1.16 (1.06, 1.27)** **0.002**	1 (ref)	1.09 (0.99, 1.21) 0.08	**1.11 (1.00, 1.23)** **0.043**
Model III	**0.94 (0.89, 0.98)** **0.007**	**1.22 (1.12, 1.33)** **<0.001**	**1.16 (1.04, 1.29)** **0.008**	1 (ref)	1.10 (0.99, 1.22) 0.08	1.07 (0.96, 1.19) 0.24
**MCV (fl)**		≤86.1	86.2–89.0	89.1–91.3	91.4–93.9	≥94.0
Model I	**1.02 (1.01, 1.02)** **<0.001**	**1.26 (1.13, 1.40)** **<0.001**	1.10 (0.99, 1.23) 0.09	1 (ref)	1.07 (0.96, 1.20) 0.21	**1.40 (1.27, 1.54)** **<0.001**
Model II	**(1.00, 1.02)** **0.007**	**1.30 (1.16, 1.45)** **<0.001**	**1.12 (1.01, 1.24)** **0.038**	1 (ref)	1.06 (0.95, 1.18) 0.33	**1.32 (1.20, 1.44)** **<0.001**
Model III	**1.02 (1.01, 1.03)** **<0.001**	**1.14 (1.02, 1.28)** **0.027**	1.09 (0.97, 1.21) 0.15	1 (ref)	1.07 (0.96, 1.19) 0.24	**1.33 (1.21, 1.46)** **<0.001**

BMI: body mass index; RCIs: red blood cell indices; MCV: mean corpuscular volume; MCH: mean corpuscular hemoglobin; MCHC: mean corpuscular hemoglobin concentration; CVD: cardiovascular disease; CKD: chronic kidney disease; COPD: chronic obstructive pulmonary disease.

### Threshold effect analysis of RCIs

**Fig 2:**
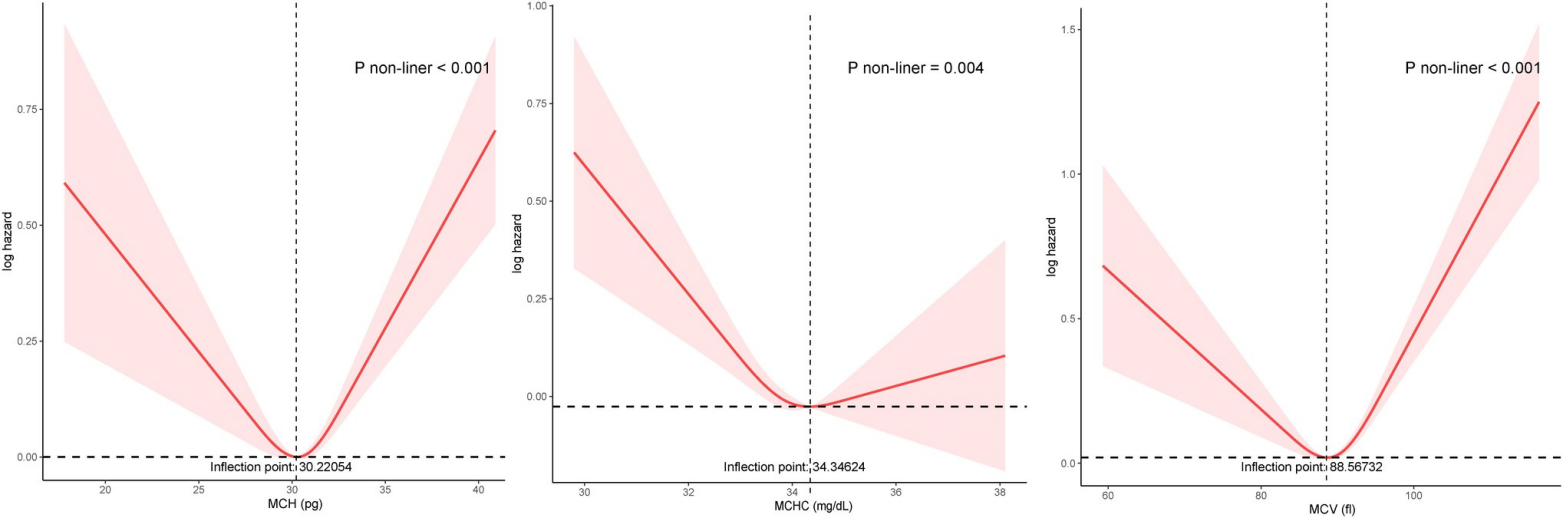
Curve fitting of the relationship between RCIs and all-cause mortality.

**Fig 3:**
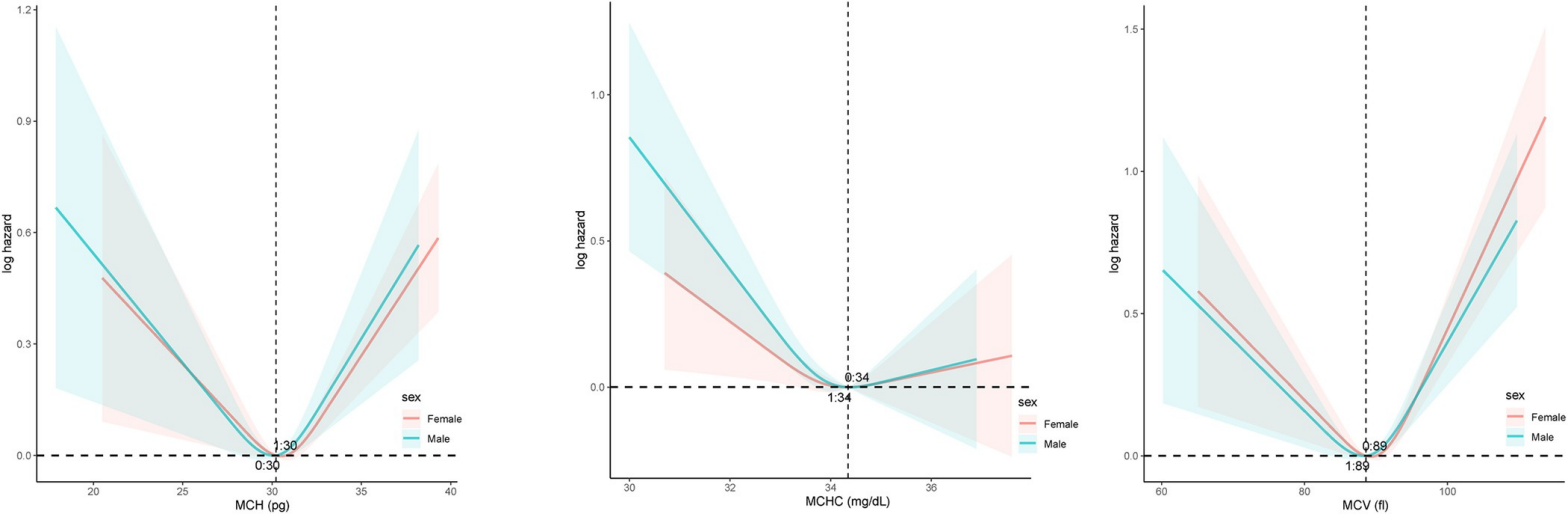
Curve fitting after sex stratification of the relationship between RCIs and all-cause mortality.

The curve fitting revealed a nonlinear relationship between the three RCIs and all-cause mortality (Fig 2). Among them, the curves of the MCH and MCV groups exhibited a "U" shape, and the MCHC was an "L" shape. The trend of the fitted curves remains stable after gender stratification (Fig 3). In addition, regarding cardiovascular mortality, the MCV curve was U-shaped, and the MCH curve was L-shaped (S1 Fig).

We identified inflection points in the relationship between MCV, MCH, and MCHC and all-cause mortality as 88.56732 fl, 30.22054 pg, 34.34624 g/dl. Based on the inflection points, we performed threshold effect analyses (Table 4). To the left of the inflection point of the MCH (< 30.22054 pg), the hazard ratio is 0.95 (95% CI: 0.92–0.98, *P* = 0.003). On the right of the inflection point are 1.08 (1.04–1.12, *P* <0.001). MCV was found to be negatively related to all-cause mortality when MCV < 88.56732 fl (adjust HR = 0.99, 95% CI = 0.97–1.00, *P* = 0.041). With MCV > 88.56732 fl, MCV was observed to be positively correlated with all-cause mortality (adjust HR = 1.05, 95% CI = 1.04–1.06, *P* <0.001). If MCHC was less than the inflection point (34.34624 g/dl), it was negatively associated with the risk of death (adjust HR = 0.88, 95% CI = 0.83–0.93, *P* <0.001). However, no significant relationship was detected when MCHC was greater than the inflection point. S4 Table presented threshold effect analyses between RCIs and cardiovascular mortality. The effect size was 0.97 and 1.04 for MCV to the left and right of the inflection point, respectively. MCH was negatively associated with cardiovascular mortality only when greater than the inflection point (adjust HR = 0.91, 95% CI = 0.85–0.98, *P* = 0.011). Whereas MCHC less than the inflection point was in a negative correlation with cardiovascular mortality (adjust HR = 0.85, 95% CI = 0.77–0.94, *P* = 0.001). Still, no association was found between high MCHC and cardiovascular mortality.

**Table 4:** Threshold effect analysis of relationship of RCIs on all-cause mortality.

	Adjusted HR (95% CI)	*P*
**MCH (pg)**		
Inflection point	30.22054	
MCH < 30.22054	0.95 (0.92, 0.98)	**0.003**
MCH > 30.22054	1.08 (1.04, 1.12)	**<0.001**
Log likelihood ratio	**<0.001**	
**MCHC (g/dl)**		
Inflection point	34.34624	
MCHC < 34.34624	0.88 (0.83, 0.93)	**<0.001**
MCHC > 34.34624	1.02 (0.79, 1.30)	0.881
Log likelihood ratio	0.297	
**MCV (fl)**		
Inflection point	88.56732	
MCV < 88.56732	0.99 (0.97,1.00)	**0.041**
MCV > 88.56732	1.05 (1.04,1.06)	**<0.001**
Log likelihood ratio	**<0.001**	

Adjusted for Model III.

BMI: body mass index; RCIs: red blood cell indices; MCV: mean corpuscular volume; MCH: mean corpuscular hemoglobin; MCHC: mean corpuscular hemoglobin concentration; CVD: cardiovascular disease; CKD: chronic kidney disease; COPD: chronic obstructive pulmonary disease.

### Sensitivity analysis

After excluding anemic patients and those who died within two years, we performed threshold effect analyses for RCIs and all-cause mortality (S5 and S6 Tables). The results remain stable. S7 Table showed that the relationship between the three RCIs and all-cause mortality was essentially stable across subgroups. In addition, it is important to note that in the CVD and COPD populations, the interaction effect values with MCH were 0.01 and 0.02, respectively. In terms of all-cause mortality, there existed an interaction between hypertension and MCV (*p* for interaction = 0.03), and an interaction between CVD and MCHC (*p* for interaction = 0.03).

## Discussion

The primary findings of this study are: (i) RCIs (MCH, MCHC, and MCV) were related to the risk of all-cause mortality in the general population. (ii) There were nonlinear relationships between the three RCIs and all-cause mortality. (iii) MCV and MCH were nonlinearly associated with the risk of cardiovascular mortality.

A prospective cohort study (n = 403) in an elderly population showed that MCV, MCH, and MCHC were unrelated to all-cause mortality in females, whereas males demonstrated a correlation [23]. This is different from some of our findings. First, it has a relatively small sample size. Second, the criteria for grouping the same RCI of different sexes in the experiment differed. Finally, from our inflection point analysis, inflection points were included within the control or second-class subgroups in the prospective study. These may be the reasons for the biased results. Further, similar to our findings, a retrospective study found a U-shaped relationship between MCH, MCV, and acute mortality (1 month) in emergency patients [24]. Another retrospective study discovered a "J" shaped relationship between MCV and in-hospital mortality in cardiac intensive care unit patients [25]. The above studies are related to short-term mortality; further studies on long-term and cause-specific mortality are lacking.

A study identified a 119% higher mortality in people with stage 3–5 CKD with high MCV (≥90.8 fl) than in the low MCV (<90.8 fl) group [26]. Zhang noted that high MCV and MCH increased the incidence of adverse cardiovascular events in nonanemic patients with acute coronary syndromes [27]. High MCV manifests many underlying disorders (bone marrow dysfunction, malnutrition, organ dysfunction, endothelial dysfunction), and these underlying problems eventually develop into a poor prognosis [28–31]. Even studies have pointed out that macrocytosis caused by elevated MCV seems to affect the Cardiac Intensive Care Unit population more than microcytosis caused by elevated RDW [25]. Huang’s study indicated that abnormalities in RBC and HCT, which determine MCV values, are correlated with the risk of developing hypertension [32]. MCV abnormalities reduce NO production by endothelial cells leading to vasoconstriction, increased blood viscosity leading to arterial stiffness and ultimately an increase in blood pressure [33]. The subgroup analyses in this paper also suggested that the relationship between MCV and mortality in hypertension needs to be further explored. Elevated MCV and MCH are a sign of macrocytosis [34]. Erythrocyte deformability increased with aging in healthy females and was significantly and positively linked to MCV and MCH [35]. The morphological dysfunction of erythrocytes is followed by impairment of extracellular antioxidant capacity, redox imbalance, and changes in cellular homeostasis [36]. However, macrocytes can generally persist in circulation for longer periods without being remodeled, which can exacerbate the process of obstructing their microcirculatory flow [37].

There was no relationship between high levels of MCHC and mortality in our study. Meanwhile, MCHC was insignificant to the right of the inflection point in the threshold effects analysis. It may be due to the smaller size of the red blood cells and their higher hemoglobin content, which allows the cells to pass through the tiny capillaries more easily [38]. Decreased MCHC can roughly represent the presence of hypochromia, which is common in patients with moderate and severe anemia [39, 40]. We detected a significant negative association with the risk of cardiovascular mortality when MCHC was less than the inflection point (34.34624 g/dl). In a retrospective cohort study, low levels of MCHC are linked to poor prognosis in individuals with acute pulmonary embolism [32]. Studies have indicated a correlation with the incidence of non-atherosclerotic cardiovascular disease when MCHC is <32 g/dl [41]. Low MCHC reduces organ oxygenation after long-term development. Lower MCHC mirrors greater cardiac output demand and higher afterload, resulting in increased left ventricle wall thickness and left atrium internal diameter [39, 42]. It also explained the emergence of MCHC interactions in the CVD population (*p* for interaction = 0.03).

This study has several strengths. The study sample was adequate and had a long follow-up period. Besides, adjustments were made to the extent possible based on the major confounders that may affect the final results. The experiment still has limitations. First, although we included key indicators and comorbidities, there may still be other unmeasured confounding factors. Second, because comorbidities are self-reported, we cannot avoid potential cognitive errors. Third, the NHANES data are based on a survey of a representative sample of noninstitutionalized residents in the United States, which has single-center limitations. Therefore, the results of this study need to be validated with more cross-national or cross-regional clinical data. Last, the experimental procedure was measured only once for the participants, so repeated measurements of the data for reanalysis were incorporated into our future research directions.

## Conclusion

This cohort study demonstrated that RCIs (MCH, MCHC, and MCV) were associated with a risk of long-term all-cause mortality in the general population. MCH and MCV have a U-shaped relationship with all-cause mortality, and MCHC has an L-shape. Furthermore, regarding cardiovascular mortality, the MCV curve was U-shaped, and the MCH curve was L-shaped. People need to focus on RCIs, which can contribute to improving patient prognosis in the clinic. Further studies are required in order to validate our findings and elucidate the underlying mechanisms between the three RCIs and mortality risk.

## Supporting information

S1 Table Baseline characteristic of the study population (based on MCH quintiles). (DOCX)

S2 Table Baseline characteristic of the study population (based on MCHC quintiles). (DOCX)

S3 Table Baseline characteristic of the study population (based on MCV quintiles). (DOCX)

S4 Table Threshold effect analysis of relationship of RCIs on cardiovascular mortality. (DOCX)

S5 Table Threshold effect analysis of relationship of RCIs on all-cause mortality after exclusion of anemic patients (n = 19672). (DOCX)

S6 Table Threshold effect analysis of relationship of RCIs on all-cause mortality after excluding patients died within two years (n = 20740). (DOCX)

S7 Table Subgroup analyses of RCIs and all-cause mortality. (DOCX)

S1 Fig Curve fitting of the relationship between RCIs and cardiovascular mortality. (TIF)

S1 Appendix Additional document. (DOCX)

S2 Appendix Original data. (XLSX)
