# Supplementary material for: Nonlinear relationship of red blood cell indices (MCH, MCHC, and MCV) with all-cause and cardiovascular mortality: A cohort study in U.S. adults
Source: PLoS One. 2024 Aug 2;19(8):e0307609. doi: 10.1371/journal.pone.0307609 (PMC11296621; doi:10.1371/journal.pone.0307609)
Supplement: S1 Table — (DOCX) [file pone.0307609.s001.docx]

**Table S1 Baseline characteristic of the study population (based on MCH quintiles)**


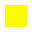


| **Variables** | **Q1**  **≤29.1**  **(n = 4451)** | **Q2**  **29.2-30.3**  **(n = 4626)** | **Q3**  **30.4-31.1**  **(n = 3821)** | **Q4**  **31.2-32.1**  **(n = 4225)** | **Q5**  **≥32.2**  **(n = 4080)** | ***P* value** |
| --- | --- | --- | --- | --- | --- | --- |
| **Age, years** | 46.9 ± 17.6 | 48.4 ± 18.1 | 50.1 ± 18.3 | 51.7 ± 18.3 | 55.7 ± 18.1 | < 0.001 |
| **Genders, %** |  |  |  |  |  | < 0.001 |
| Female | 2612 (58.7) | 2283 (49.4) | 1823 (47.7) | 1986 (47.0) | 1820 (44.6) |  |
| Male | 1839 (41.3) | 2343 (50.6) | 1998 (52.3) | 2239 (53.0) | 2260 (55.4) |  |
| **Ethnicity, %** |  |  |  |  |  | < 0.001 |
| Non-Hispanic White | 1347 (30.3) | 2254 (48.7) | 2055 (53.8) | 2407 (57.0) | 2571 (63.0) |  |
| Mexican American | 855 (19.2) | 1043 (22.5) | 891 (23.3) | 900 (21.3) | 735 (18.0) |  |
| Non-Hispanic Black | 1745 (39.2) | 885 (19.1) | 545 (14.3) | 520 (12.3) | 460 (11.3) |  |
| Other Race | 504 (11.3) | 444 (9.6) | 330 (8.6) | 398 (9.4) | 314 (7.7) |  |
| **Education, %** |  |  |  |  |  | 0.171 |
| <High school diploma | 600 (13.5) | 704 (15.2) | 569 (14.9) | 599 (14.2) | 588 (14.4) |  |
| Completed high school | 1872 (42.1) | 1837 (39.7) | 1514 (39.6) | 1688 (40.0) | 1674 (41.0) |  |
| ≥ College | 1979 (44.5) | 2085 (45.1) | 1738 (45.5) | 1938 (45.9) | 1818 (44.6) |  |
| **BMI, %** |  |  |  |  |  | < 0.001 |
| <25 | 1237 (26.7) | 1206 (31.6) | 1455 (34.4) | 1673 (41.0) | 982 (22.1) |  |
| 25-30 | 1666 (36.0) | 1423 (37.2) | 1614 (38.2) | 1415 (34.7) | 1417 (31.8) |  |
| >30 | 1723 (37.2) | 1192 (31.2) | 1156 (27.4) | 992 (24.3) | 2052 (46.1) |  |
| **Smoke, %** |  |  |  |  |  | < 0.001 |
| Never smoker | 2717 (61.0) | 2579 (55.8) | 1972 (51.6) | 2005 (47.5) | 1558 (38.2) |  |
| Former smoker | 1017 (22.8) | 1188 (25.7) | 1037 (27.1) | 1167 (27.6) | 1175 (28.8) |  |
| Current smoker | 717 (16.1) | 859 (18.6) | 812 (21.3) | 1053 (24.9) | 1347 (33.0) |  |
| **Comorbidities, %** |  |  |  |  |  |  |
| CVD | 483 (10.9) | 458 (9.9) | 409 (10.7) | 504 (11.9) | 608 (14.9) | < 0.001 |
| Hypertension | 1916 (43.0) | 1857 (40.1) | 1547 (40.5) | 1736 (41.1) | 1845 (45.2) | < 0.001 |
| Hyperlipidemia | 1157 (26.0) | 1162 (25.1) | 1008 (26.4) | 1116 (26.4) | 1098 (26.9) | 0.395 |
| Diabetes | 858 (19.3) | 741 (16.0) | 568 (14.9) | 508 (12.0) | 581 (14.2) | < 0.001 |
| CKD | 872 (19.6) | 868 (18.8) | 667 (17.5) | 779 (18.4) | 953 (23.4) | < 0.001 |
| COPD | 147 (3.3) | 141 (3.0) | 136 (3.6) | 186 (4.4) | 228 (5.6) | < 0.001 |
| Cancer | 283 (6.4) | 372 (8.0) | 324 (8.5) | 400 (9.5) | 516 (12.6) | < 0.001 |
| Anemia | 814 (18.3) | 215 (4.6) | 139 (3.6) | 150 (3.6) | 213 (5.2) | < 0.001 |
| **Mortality, %** |  |  |  |  |  |  |
| All-cause | 893 (20.1) | 939 (20.3) | 873 (22.8) | 1024 (24.2) | 1444 (35.4) | < 0.001 |
| Cardiovascular | 261 (5.9) | 240 (5.2) | 228 (6.0) | 273 (6.5) | 347 (8.5) | < 0.001 |

BMI: body mass index; MCV: mean corpuscular volume; MCH: mean corpuscular hemoglobin; MCHC: mean corpuscular hemoglobin concentration; CVD: cardiovascular disease; CKD: chronic kidney disease; COPD: chronic obstructive pulmonary disease.
