# Supplementary material for: Nonlinear relationship of red blood cell indices (MCH, MCHC, and MCV) with all-cause and cardiovascular mortality: A cohort study in U.S. adults
Source: PLoS One. 2024 Aug 2;19(8):e0307609. doi: 10.1371/journal.pone.0307609 (PMC11296621; doi:10.1371/journal.pone.0307609)
Supplement: S2 Table — (DOCX) [file pone.0307609.s002.docx]

**Table S2 Baseline characteristic of the study population (based on MCHC quintiles)**


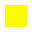


| **Variables** | **Q1**  **≤33.3**  **(n = 5330)** | **Q2**  **33.4-33.7**  **(n = 3264)** | **Q3**  **33.8-34.2**  **(n = 5428)** | **Q4**  **34.3-34.7**  **(n = 3544)** | **Q5**  **≥34.8**  **(n = 3637)** | ***p* value** |
| --- | --- | --- | --- | --- | --- | --- |
| **Age, years** | 51.5 ± 18.7 | 52.3 ± 18.7 | 50.7 ± 18.5 | 49.6 ± 17.9 | 47.8 ± 17.3 | < 0.001 |
| **Genders, %** |  |  |  |  |  | < 0.001 |
| Female | 2701 (50.7) | 1632 (50.0) | 2719 (50.1) | 1805 (50.9) | 1667 (45.8) |  |
| Male | 2629 (49.3) | 1632 (50.0) | 2709 (49.9) | 1739 (49.1) | 1970 (54.2) |  |
| **Ethnicity, %** |  |  |  |  |  | < 0.001 |
| Non-Hispanic White | 1901 (35.7) | 1605 (49.2) | 2986 (55.0) | 2090 (59.0) | 2052 (56.4) |  |
| Mexican American | 926 (17.4) | 710 (21.8) | 1153 (21.2) | 762 (21.5) | 873 (24.0) |  |
| Non-Hispanic Black | 2037 (38.2) | 676 (20.7) | 790 (14.6) | 385 (10.9) | 267 (7.3) |  |
| Other Race | 466 (8.7) | 273 (8.4) | 499 (9.2) | 307 (8.7) | 445 (12.2) |  |
| **Education, %** |  |  |  |  |  | 0.011 |
| <High school diploma | 800 (15.0) | 501 (15.3) | 765 (14.1) | 510 (14.4) | 484 (13.3) |  |
| Completed high school | 2188 (41.1) | 1319 (40.4) | 2209 (40.7) | 1352 (38.1) | 1517 (41.7) |  |
| ≥ College | 2342 (43.9) | 1444 (44.2) | 2454 (45.2) | 1682 (47.5) | 1636 (45.0) |  |
| **BMI, %** |  |  |  |  |  | < 0.001 |
| <25 | 1561 (29.3) | 1023 (31.3) | 1762 (32.5) | 1134 (32.0) | 1073 (29.5) |  |
| 25-30 | 1812 (34.0) | 1147 (35.1) | 1949 (35.9) | 1254 (35.4) | 1373 (37.8) |  |
| >30 | 1957 (36.7) | 1094 (33.5) | 1717 (31.6) | 1156 (32.6) | 1191 (32.7) |  |
| **Smoke, %** |  |  |  |  |  | < 0.001 |
| Never smoker | 2896 (54.3) | 1682 (51.5) | 2726 (50.2) | 1764 (49.8) | 1763 (48.5) |  |
| Former smoker | 1404 (26.3) | 863 (26.4) | 1493 (27.5) | 921 (26.0) | 903 (24.8) |  |
| Current smoker | 1030 (19.3) | 719 (22.0) | 1209 (22.3) | 859 (24.2) | 971 (26.7) |  |
| **Comorbidities, %** |  |  |  |  |  |  |
| CVD | 724 (13.6) | 393 (12.0) | 607 (11.2) | 374 (10.6) | 364 (10.0) | < 0.001 |
| Hypertension | 2469 (46.3) | 1428 (43.8) | 2195 (40.4) | 1391 (39.2) | 1418 (39.0) | < 0.001 |
| Hyperlipidemia | 1568 (29.4) | 837 (25.6) | 1377 (25.4) | 893 (25.2) | 866 (23.8) | < 0.001 |
| Diabetes | 929 (17.4) | 512 (15.7) | 769 (14.2) | 501 (14.1) | 545 (15.0) | < 0.001 |
| CKD | 1192 (22.4) | 695 (21.3) | 1044 (19.2) | 602 (17) | 606 (16.7) | < 0.001 |
| COPD | 199 (3.7) | 131 (4.0) | 197 (3.6) | 132 (3.7) | 179 (4.9) | 0.020 |
| Cancer | 449 (8.4) | 312 (9.6) | 515 (9.5) | 315 (8.9) | 304 (8.4) | 0.147 |
| Anemia | 818 (15.3) | 198 (6.1) | 244 (4.5) | 137 (3.9) | 134 (3.7) | < 0.001 |
| **Mortality, %** |  |  |  |  |  |  |
| All-cause | 1540 (28.9) | 928 (28.4) | 1283 (23.6) | 776 (21.9) | 646 (17.8) | < 0.001 |
| Cardiovascular | 442 (8.3) | 242 (7.4) | 331 (6.1) | 193 (5.4) | 141 (3.9) | < 0.001 |

BMI: body mass index; MCV: mean corpuscular volume; MCH: mean corpuscular hemoglobin; MCHC: mean corpuscular hemoglobin concentration; CVD: cardiovascular disease; CKD: chronic kidney disease; COPD: chronic obstructive pulmonary disease.
