# Supplementary material for: Nonlinear relationship of red blood cell indices (MCH, MCHC, and MCV) with all-cause and cardiovascular mortality: A cohort study in U.S. adults
Source: PLoS One. 2024 Aug 2;19(8):e0307609. doi: 10.1371/journal.pone.0307609 (PMC11296621; doi:10.1371/journal.pone.0307609)
Supplement: S3 Table — (DOCX) [file pone.0307609.s003.docx]

**Table S3 Baseline characteristic of the study population (based on MCV quintiles)**


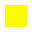


| **Variables** | **Q1**  **≤86.1**  **(n = 4301)** | **Q2**  **86.2-89.0**  **(n = 4235)** | **Q3**  **89.1-91.3**  **(n = 4256)** | **Q4**  **91.4-93.9**  **(n = 4291)** | **Q5**  **≥94.0**  **(n = 4120)** | ***p* value** |
| --- | --- | --- | --- | --- | --- | --- |
| **Age, years** | 45.7 ± 17.0 | 47.4 ± 17.8 | 49.5 ± 18.0 | 52.6 ± 18.3 | 57.3 ± 18.2 | < 0.001 |
| **Genders, %** |  |  |  |  |  | < 0.001 |
| Female | 2523 (58.7) | 2114 (49.9) | 2024 (47.6) | 2038 (47.5) | 1825 (44.3) |  |
| Male | 1778 (41.3) | 2121 (50.1) | 2232 (52.4) | 2253 (52.5) | 2295 (55.7) |  |
| **Ethnicity, %** |  |  |  |  |  | < 0.001 |
| Non-Hispanic White | 1397 (32.5) | 2078 (49.1) | 2201 (51.7) | 2426 (56.5) | 2532 (61.5) |  |
| Mexican American | 889 (20.7) | 959 (22.6) | 997 (23.4) | 903 (21) | 676 (16.4) |  |
| Non-Hispanic Black | 1480 (34.4) | 771 (18.2) | 675 (15.9) | 602 (14) | 627 (15.2) |  |
| Other Race | 535 (12.4) | 427 (10.1) | 383 (9) | 360 (8.4) | 285 (6.9) |  |
| **Education, %** |  |  |  |  |  | 0.187 |
| <High school diploma | 576 (13.4) | 598 (14.1) | 646 (15.2) | 635 (14.8) | 605 (14.7) |  |
| Completed high school | 1802 (41.9) | 1696 (40) | 1697 (39.9) | 1698 (39.6) | 1692 (41.1) |  |
| ≥ College | 1923 (44.7) | 1941 (45.8) | 1913 (44.9) | 1958 (45.6) | 1823 (44.2) |  |
| **BMI, %** |  |  |  |  |  | < 0.001 |
| <25 | 892 (20.7) | 1102 (26) | 1317 (30.9) | 1509 (35.2) | 1733 (42.1) |  |
| 25-30 | 1388 (32.3) | 1526 (36) | 1570 (36.9) | 1623 (37.8) | 1428 (34.7) |  |
| >30 | 2021 (47) | 1607 (37.9) | 1369 (32.2) | 1159 (27) | 959 (23.3) |  |
| **Smoke, %** |  |  |  |  |  | < 0.001 |
| Never smoker | 2671 (62.1) | 2356 (55.6) | 2206 (51.8) | 2009 (46.8) | 1589 (38.6) |  |
| Former smoker | 954 (22.2) | 1063 (25.1) | 1129 (26.5) | 1191 (27.8) | 1247 (30.3) |  |
| Current smoker | 676 (15.7) | 816 (19.3) | 921 (21.6) | 1091 (25.4) | 1284 (31.2) |  |
| **Comorbidities, %** |  |  |  |  |  |  |
| CVD | 419 (9.7) | 400 (9.4) | 429 (10.1) | 553 (12.9) | 661 (16) | < 0.001 |
| Hypertension | 1788 (41.6) | 1667 (39.4) | 1668 (39.2) | 1830 (42.6) | 1948 (47.3) | < 0.001 |
| Hyperlipidemia | 1091 (25.4) | 1030 (24.3) | 1104 (25.9) | 1147 (26.7) | 1169 (28.4) | < 0.001 |
| Diabetes | 804 (18.7) | 703 (16.6) | 581 (13.7) | 609 (14.2) | 559 (13.6) | < 0.001 |
| CKD | 785 (18.3) | 746 (17.6) | 739 (17.4) | 828 (19.3) | 1041 (25.3) | < 0.001 |
| COPD | 137 (3.2) | 125 (3) | 163 (3.8) | 184 (4.3) | 229 (5.6) | < 0.001 |
| Cancer | 259 (6) | 311 (7.3) | 341 (8) | 454 (10.6) | 530 (12.9) | < 0.001 |
| Anemia | 767 (17.8) | 208 (4.9) | 167 (3.9) | 154 (3.6) | 235 (5.7) | < 0.001 |
| **Mortality, %** |  |  |  |  |  |  |
| All-cause | 726 (16.9) | 776 (18.3) | 898 (21.1) | 1144 (26.7) | 1629 (39.5) | < 0.001 |
| Cardiovascular | 205 (4.8) | 205 (4.8) | 228 (5.4) | 314 (7.3) | 397 (9.6) | < 0.001 |

BMI: body mass index; MCV: mean corpuscular volume; MCH: mean corpuscular hemoglobin; MCHC: mean corpuscular hemoglobin concentration; CVD: cardiovascular disease; CKD: chronic kidney disease; COPD: chronic obstructive pulmonary disease.
