# Supplementary material for: Nonlinear relationship of red blood cell indices (MCH, MCHC, and MCV) with all-cause and cardiovascular mortality: A cohort study in U.S. adults
Source: PLoS One. 2024 Aug 2;19(8):e0307609. doi: 10.1371/journal.pone.0307609 (PMC11296621; doi:10.1371/journal.pone.0307609)
Supplement: S4 Table — (DOCX) [file pone.0307609.s004.docx]

**Table S4 Threshold effect analysis of relationship of RCIs on cardiovascular mortality**

|  | **Cardiovascular mortality** | |
| --- | --- | --- |
|  | **HR (95% CI)** | ***p* value** |
| **MCH (pg)** |  |  |
| Continuous | 0.98 (0.95,1.02) | 0.280 |
| Inflection point | 30.22054 |  |
| MCH < 30.22054 | 1.03 (0.98,1.08) | 0.211 |
| MCH > 30.22054 | 0.91 (0.85,0.98) | 0.011 |
| Log likelihood ratio | 0.012 |  |
| **MCHC (g/dl)** |  |  |
| Continuous | 0.87 (0.80,0.94) | <0.001 |
| Inflection point | 34.34624 |  |
| MCHC < 34.34624 | 0.85 (0.77,0.94) | 0.001 |
| MCHC > 34.34624 | 0.95 (0.69,1.31) | 0.769 |
| Log likelihood ratio | 0.674 |  |
| **MCV (fl)** |  |  |
| Continuous | 1.00 (0.99,1.02) | 0.735 |
| Inflection point | 88.56732 |  |
| MCV < 88.56732 | 0.97 (0.94,1.00) | 0.047 |
| MCV > 88.56732 | 1.04 (1.01,1.06) | <0.001 |
| Log likelihood ratio | 0.001 |  |

Adjusted for Model III.

BMI: body mass index; RCIs: red blood cell indices; MCV: mean corpuscular volume; MCH: mean corpuscular hemoglobin; MCHC: mean corpuscular hemoglobin concentration; CVD: cardiovascular disease; CKD: chronic kidney disease; COPD: chronic obstructive pulmonary disease.
