# Supplementary material for: Nonlinear relationship of red blood cell indices (MCH, MCHC, and MCV) with all-cause and cardiovascular mortality: A cohort study in U.S. adults
Source: PLoS One. 2024 Aug 2;19(8):e0307609. doi: 10.1371/journal.pone.0307609 (PMC11296621; doi:10.1371/journal.pone.0307609)
Supplement: S6 Table — (DOCX) [file pone.0307609.s006.docx]

**Table S6 Threshold effect analysis of relationship of RCIs on all-cause mortality after excluding patients died within two years (n=20740)**

|  | **Adjusted HR (95% CI)** | ***p* value** |
| --- | --- | --- |
| **MCH (pg)** |  |  |
| Continuous | 1.03 (1.01,1.05) | 0.006 |
| Inflection point | 30.22054 |  |
| MCH < 30.22054 | 0.95 (0.91,0.99) | 0.006 |
| MCH > 30.22054 | 1.08 (1.03,1.12) | <0.001 |
| Log likelihood ratio | <0.001 |  |
| **MCHC (g/dl)** |  |  |
| Continuous | 0.96 (0.91,1.01) | 0.102 |
| Inflection point | 34.34624 |  |
| MCHC < 34.34624 | 0.91 (0.85,0.97) | 0.002 |
| MCHC > 34.34624 | 1.02 (0.78,1.33) | 0.895 |
| Log likelihood ratio | 0.441 |  |
| **MCV (fl)** |  |  |
| Continuous | 1.02 (1.01,1.03) | <0.001 |
| Inflection point | 88.56732 |  |
| MCV < 88.56732 | 0.99 (0.97,1.00) | 0.057 |
| MCV > 88.56732 | 1.05 (1.03,1.06) | <0.001 |
| Log likelihood ratio | <0.001 |  |

Adjusted for Model III.

BMI: body mass index; RCIs: red blood cell indices; MCV: mean corpuscular volume; MCH: mean corpuscular hemoglobin; MCHC: mean corpuscular hemoglobin concentration; CVD: cardiovascular disease; CKD: chronic kidney disease; COPD: chronic obstructive pulmonary disease.
