# Supplementary material for: Nonlinear relationship of red blood cell indices (MCH, MCHC, and MCV) with all-cause and cardiovascular mortality: A cohort study in U.S. adults
Source: PLoS One. 2024 Aug 2;19(8):e0307609. doi: 10.1371/journal.pone.0307609 (PMC11296621; doi:10.1371/journal.pone.0307609)
Supplement: S7 Table — (DOCX) [file pone.0307609.s007.docx]

**Table S7 Subgroup analyses of RCIs and all-cause mortality**

|  | **MCV** | | | **MCH** | | | **MCHC** | | |
| --- | --- | --- | --- | --- | --- | --- | --- | --- | --- |
| **Subgroup** | **HR (95% CI)** | ***p*** | ***p* for**  **interaction** | **HR (95% CI)** | ***p*** | ***p* for**  **interaction** | **HR (95% CI)** | ***p*** | ***p* for interaction** |
| **Age** |  |  | 0.51 |  |  | 0.93 |  |  | 0.38 |
| >50 | 1.02(1.01,1.02) | <0.01 |  | 1.02(1.00,1.04) | 0.04 |  | 0.92(0.88,0.97) | <0.01 |  |
| ≤50 | 1.02(0.99,1.05) | 0.12 |  | 1.04(0.97,1.12) | 0.27 |  | 0.97(0.80,1.18) | 0.76 |  |
| **Genders** |  |  | 0.63 |  |  | 0.5 |  |  | 0.16 |
| Male | 1.02(1.01,1.03) | <0.01 |  | 1.03(1.01,1.06) | 0.01 |  | 0.96(0.89,1.03) | 0.24 |  |
| Female | 1.01(1.00,1.02) | 0.03 |  | 1.01(0.98,1.04) | 0.47 |  | 0.90(0.84,0.96) | <0.01 |  |
| **Ethnicity** |  |  | 0.23 |  |  | 0.56 |  |  | 0.6 |
| White | 1.02(1.01,1.03) | <0.01 |  | 1.03(1.00,1.05) | 0.03 |  | 0.94(0.89,0.99) | 0.01 |  |
| Black | 1.02(1.00,1.03) | 0.02 |  | 1.03(1.00,1.07) | 0.09 |  | 0.95(0.87,1.05) | 0.32 |  |
| Mexican | 1.02(1.00,1.05) | 0.10 |  | 1.03(0.97,1.10) | 0.34 |  | 0.96(0.85,1.08) | 0.48 |  |
| Other | 1.01(0.99,1.04) | 0.34 |  | 1.04(0.97,1.13) | 0.27 |  | 1.10(0.80,1.51) | 0.58 |  |
| **Education** |  |  | 0.7 |  |  | 0.61 |  |  | 0.2 |
| ≥College | 1.02(1.00,1.03) | 0.02 |  | 1.02(0.99,1.05) | 0.30 |  | 0.92(0.84,1.01) | 0.10 |  |
| =high school | 1.02(1.01,1.03) | <0.01 |  | 1.03(1.01,1.06) | 0.02 |  | 0.93(0.88,0.99) | 0.01 |  |
| <high school | 1.01(0.99,1.02) | 0.27 |  | 1.01(0.97,1.04) | 0.70 |  | 0.94(0.87,1.01) | 0.09 |  |
| **BMI** |  |  | 0.57 |  |  | 0.18 |  |  | 0.19 |
| <25 | 1.00(0.99,1.02) | 0.55 |  | 1.01(0.97,1.04) | 0.74 |  | 0.98(0.88,1.08) | 0.65 |  |
| 25-30 | 1.03(1.02,1.04) | <0.01 |  | 1.06(1.03,1.09) | <0.01 |  | 0.97(0.90,1.05) | 0.52 |  |
| >30 | 1.01(1.00,1.03) | 0.06 |  | 1.00(0.98,1.03) | 0.75 |  | 0.89(0.83,0.96) | <0.01 |  |
| **Smoke** |  |  | 0.12 |  |  | 0.06 |  |  | 0.06 |
| Never | 1.01(1.00,1.02) | 0.06 |  | 1.01(0.98,1.04) | 0.41 |  | 0.92(0.86,0.98) | 0.02 |  |
| Former | 1.01(1.00,1.02) | 0.01 |  | 1.01(0.99,1.04) | 0.37 |  | 0.93(0.85,1.03) | 0.17 |  |
| Current | 1.02(1.00,1.04) | 0.02 |  | 1.04(1.00,1.08) | 0.08 |  | 0.94(0.87,1.01) | 0.11 |  |
| **CKD** |  |  | 0.42 |  |  | 0.2 |  |  | 0.56 |
| NO | 1.02(1.00,1.03) | 0.01 |  | 1.03(1.00,1.06) | 0.04 |  | 0.95(0.90,1.01) | 0.13 |  |
| Yes | 1.02(1.01,1.03) | <0.01 |  | 1.01(0.99,1.04) | 0.24 |  | 0.91(0.84,0.98) | 0.01 |  |
| **Hypertension** |  |  | **0.03** |  |  | 0.19 |  |  | 0.55 |
| NO | 1.03(1.01,1.04) | <0.01 |  | 1.03(1.00,1.07) | 0.03 |  | 0.97(0.87,1.07) | 0.53 |  |
| Yes | 1.01(1.00,1.02) | 0.01 |  | 1.01(0.99,1.04) | 0.27 |  | 0.92(0.87,0.97) | <0.01 |  |
| **CVD** |  |  | 0.16 |  |  | **0.01** |  |  | **0.03** |
| NO | 1.02(1.01,1.03) | <0.01 |  | 1.03(1.01,1.06) | <0.01 |  | 0.97(0.91,1.03) | 0.34 |  |
| Yes | 1.02(1.00,1.03) | 0.01 |  | 1.00(0.98,1.03) | 0.84 |  | 0.86(0.80,0.93) | <0.01 |  |
| **DM** |  |  | 0.66 |  |  | 0.86 |  |  | 0.27 |
| NO | 1.02(1.01,1.02) | <0.01 |  | 1.02(1.00,1.04) | 0.06 |  | 0.92(0.87,0.98) | 0.01 |  |
| Yes | 1.02(1.01,1.03) | <0.01 |  | 1.03(1.00,1.07) | 0.07 |  | 0.96(0.86,1.06) | 0.39 |  |
| **COPD** |  |  | 0.06 |  |  | **0.02** |  |  | 0.5 |
| NO | 1.02(1.01,1.03) | <0.01 |  | 1.03(1.01,1.06) | 0.002 |  | 0.94(0.89,1.00) | 0.04 |  |
| Yes | 0.99(0.96,1.01) | 0.36 |  | 0.95(0.90,1.00) | 0.07 |  | 0.84(0.73,0.97) | 0.02 |  |
| **Hyperlipidemia** |  |  | 0.28 |  |  | 0.34 |  |  | 0.74 |
| NO | 1.02(1.01,1.03) | <0.01 |  | 1.02(1.00,1.04) | 0.09 |  | 0.94(0.88,0.99) | 0.02 |  |
| Yes | 1.02(1.01,1.04) | <0.01 |  | 1.04(1.01,1.07) | 0.02 |  | 0.94(0.86,1.02) | 0.15 |  |
| **Cancer** |  |  | 0.46 |  |  | 0.44 |  |  | 0.29 |
| NO | 1.02(1.01,1.03) | <0.01 |  | 1.03(1.01,1.05) | 0.01 |  | 0.94(0.89,1.00) | 0.05 |  |
| Yes | 1.01(0.99,1.03) | 0.43 |  | 1.00(0.96,1.05) | 0.94 |  | 0.91(0.84,0.99) | 0.03 |  |
| **Anemia** |  |  | 0.22 |  |  | 0.9 |  |  | 0.06 |
| NO | 1.02(1.01,1.03) | <0.01 |  | 1.02(1.00,1.04) | 0.01 |  | 0.92(0.88,0.97) | 0.001 |  |
| Yes | 1.01(1.00,1.02) | 0.15 |  | 1.03(0.99,1.06) | 0.20 |  | 1.02(0.92,1.14) | 0.67 |  |

Adjusted for Model III.

BMI: body mass index; RCIs: red blood cell indices; MCV: mean corpuscular volume; MCH: mean corpuscular hemoglobin; MCHC: mean corpuscular hemoglobin concentration; CVD: cardiovascular disease; CKD: chronic kidney disease; COPD: chronic obstructive pulmonary disease.
