# Supplementary figures and images for: Nonlinear relationship of red blood cell indices (MCH, MCHC, and MCV) with all-cause and cardiovascular mortality: A cohort study in U.S. adults
Source: PLoS One. 2024 Aug 2;19(8):e0307609. doi: 10.1371/journal.pone.0307609 (PMC11296621; doi:10.1371/journal.pone.0307609)

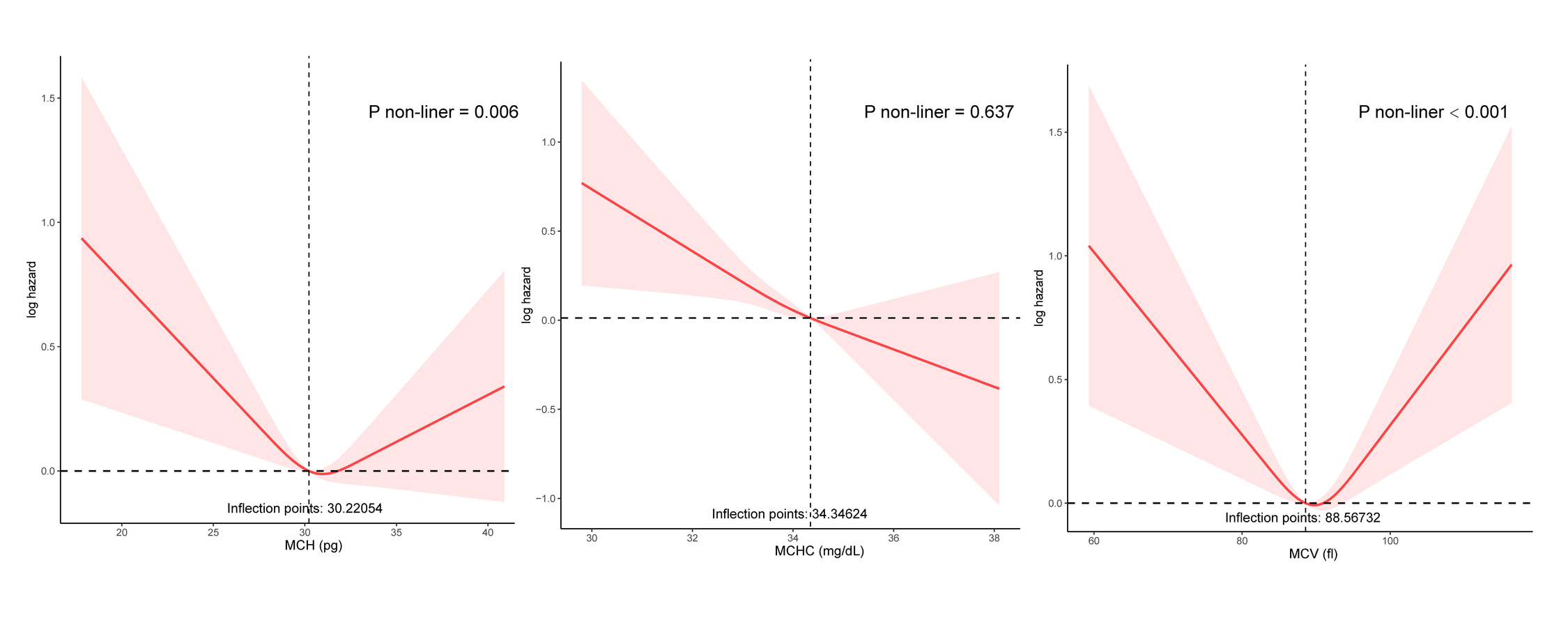

Supplement: S1 Fig — (TIF) [file pone.0307609.s008.tif]
