# Supplementary material for: Nonlinear relationship of red blood cell indices (MCH, MCHC, and MCV) with all-cause and cardiovascular mortality: A cohort study in U.S. adults
Source: PLoS One. 2024 Aug 2;19(8):e0307609. doi: 10.1371/journal.pone.0307609 (PMC11296621; doi:10.1371/journal.pone.0307609)
Supplement: S1 Appendix — (DOCX) [file pone.0307609.s009.docx]

**Definition of covariate comorbidity**

People with cardiovascular disease were defined as those who answered "yes" to the following questions: “Have you ever been told by a physician that you had coronary heart disease/ congestive heart failure/angina/a heart attack or a stroke?”^1^.

Hypertension was defined as a mean systolic blood pressure ≥ 130 mmHg, or a mean diastolic blood pressure ≥ 80 mmHg, or was taking hypertension medication or was informed of a hypertension diagnosis by a physician/health professional^2^.

Hyperlipidemia was defined as a low-density lipoprotein-cholesterol ≥130mg/dL (3.37 mmol/L), triglyceride ≥150mg/dL (1.7 mmol/L), total cholesterol ≥200mg/dL (5.18 mmol/L) or high-density lipoprotein-cholesterol <40mg/dL (1.04 mmol/L) in males and 50 mg/dL (1.30 mmol/L) in females^3^.

Diabetes was defined as the presence of one of the following conditions: diagnosed by a physician, taking glucose-lowering medication, glycosylated hemoglobin ≥6.5%, fasting blood-glucose ≥7.0 mmol/L, glucose tolerance test ≥11.1 mmol/L^4^.

Chronic kidney disease was considered present if the glomerular filtration rate <60 ml/min/1.73 m^2^ or urinary albumin-creatinine ratio ≥30 mg/g^5^.

COPD was diagnosed if any of the following criteria were met: (1) participants had a forced expiratory volume in 1 s/forced vital capacity (FVC) ratio <0.70 after inhaling β2-adrenergic bronchodilator medication; (2) participants were told to have emphysema by a doctor or other health professional and (3) participants with history of smoking and chronic bronchitis, aged 40 or over, used drugs including selective phosphodiesterase-4 inhibitors, mast cell stabilisers, leukotriene modifiers, inhaled corticosteroids^6^.

Cancer diagnoses were based on the following two questions: (i) “Have you ever been told by a doctor or other health professional that you had cancer or a malignancy of any kind?” (ii) “What kind of cancer was it and when it was diagnosed? “^7^.

Anemia was defined as hemoglobin level less than 13.0 g/dL (<130 g/L) in males and less than 12.0 g/dL (<120 g/L) in females^8^.

**Reference:**

1. Wang K, Mao Y, Lu M, et al. Association between migraine and cardiovascular disease: A cross-sectional study. *Frontiers in Cardiovascular Medicine*. 2022;9. doi:10.3389/fcvm.2022.1044465

2. Correction to: 2017 ACC/AHA/AAPA/ABC/ACPM/AGS/APhA/ASH/ASPC/NMA/PCNA Guideline for the Prevention, Detection, Evaluation, and Management of High Blood Pressure in Adults: Executive Summary: A Report of the American College of Cardiology/American Heart Association Task Force on Clinical Practice Guidelines. *Hypertension*. 2018;72(3):e33. doi:10.1161/HYP.0000000000000080

3. Jellinger PS, Handelsman Y, Rosenblit PD, et al. AMERICAN ASSOCIATION OF CLINICAL ENDOCRINOLOGISTS AND AMERICAN COLLEGE OF ENDOCRINOLOGY GUIDELINES FOR MANAGEMENT OF DYSLIPIDEMIA AND PREVENTION OF CARDIOVASCULAR DISEASE - EXECUTIVE SUMMARYComplete Appendix to Guidelines available at http://journals.aace.com. *Endocr Pract*. 2017;23(4):479-497. doi:10.4158/EP171764.GL

4. Xu Y, Wu Q. Trends in osteoporosis and mean bone density among type 2 diabetes patients in the US from 2005 to 2014. *Scientific Reports*. 2021;11. doi:10.1038/s41598-021-83263-4

5. Levey AS, Stevens LA, Schmid CH, et al. A new equation to estimate glomerular filtration rate. *Ann Intern Med*. 2009;150(9):604-612. doi:10.7326/0003-4819-150-9-200905050-00006

6. Lv JJ, Li XY, Shen YC, et al. Assessing volatile organic compounds exposure and chronic obstructive pulmonary diseases in US adults. *Front Public Health*. 2023;11:1210136. doi:10.3389/fpubh.2023.1210136

7. Ying H, Gao L, Liao N, Xu X, Yu W, Hong W. Association between niacin and mortality among patients with cancer in the NHANES retrospective cohort. *BMC Cancer*. 2022;22:1173. doi:10.1186/s12885-022-10265-4

8. Nutritional anaemias. Report of a WHO scientific group. *World Health Organization technical report series*. 1968;405. Accessed March 28, 2023. https://pubmed.ncbi.nlm.nih.gov/4975372/
